# Corrigendum to “Evaluation and Management of Skeletal Health in Celiac Disease: Position Statement”

**DOI:** 10.1155/2017/1323607

**Published:** 2017-07-02

**Authors:** Mona A. Fouda, Aliya A. Khan, Muhammad Sultan, Lorena P. Rios, Karen McAssey, David Armstrong

**Affiliations:** ^1^College of Medicine, King Saud University, Riyadh, Saudi Arabia; ^2^Department of Medicine, McMaster University, Hamilton, ON, Canada; ^3^Michael G. DeGroote School of Medicine, McMaster University, Hamilton, ON, Canada; ^4^Department of Pediatrics, McMaster University, Hamilton, ON, Canada; ^5^Division of Gastroenterology, McMaster University, Hamilton, ON, Canada

In the article titled “Evaluation and Management of Skeletal Health in Celiac Disease: Position Statement” [1], there was an error regarding the FRAX® tool, which should be clarified as follows.

The article notes: “The WHO has developed an absolute fracture risk assessment tool (‘FRAX') to estimate the 10-year fracture risk in all adults, which is based on the integration of femoral neck bone density, age and other important clinical risk factors [66].” However, the World Health Organization (WHO) did not develop, test, or endorse the FRAX tool or its recommendations [2]. The metabolic bone disease unit at the University of Sheffield that developed FRAX was a WHO Collaborating Centre from 1991 to 2010. Treatment guidelines must undergo a formal process before they can be endorsed by the WHO.
